# Vomition in the Horse—Ejection of Ascarides

**Published:** 1894-03

**Authors:** J. B. Wolstenholme

**Affiliations:** Manchester


### VOMITION IN THE HORSE—EJECTION OF ASCARIDES.

By J. B. Wolstenholme, F.R.C.V.S., Manchester.

Valuable roan cart gelding, four years old. December 1st, 1893, this horse when at work was noticed to be uneasy as though in pain at about 4 p. m., and fell down before he could be removed from the shafts. At 9 p. m. we were called in and an anodyne was given; at 10 p. m. I saw the horse myself; the patient was down evidently with abdominal pain; pulse 72, small and weak; neither blowing nor sweating; conjunctivæ somewhat injected.

The horse was quietly walked—well rugged up—for a quarter of a mile to my own premises. Ol. Lini. 15 oz., et Tr. Opii 2 oz. cum Ol. Menth. Pip. were given; this draught was repeated at 11 30 p. m.; pulse 80, small and weak; horse uneasy, lying down and rising frequently, but not at all violent. The surface of the body was very cold, although two rugs were on; stimulating liniment was well rubbed on the abdomen, the legs chafed and bandaged, the neck and head well wisped; a thick woollen neck-sheet and a similar additional rug were put on. In a short time the circulation in the skin had responded, and the surface became warmer. At 12.30 (midnight) gave chlorodyne ½ oz., et Spt. Ammon. co. 1 oz., in water. At 1.30 a. m. gave 1 oz. Spt. Ammon. co.; the pain was much relieved at this time, but the extremities had become very cold, and the surface generally was cold.

After an interval of ease lasting about one hour the pain became more marked, and continued until 6.30 a. m. The pulse at this time was 96, very small, feeble, and scarcely perceptible to the touch; conjunctivæ injected a bright red colour. A draught consisting of chlorodyne and Spt. Ammon. co. had been given every hour, and hot rugs applied to the abdomen, but the surface of the body was cold.

At 6.45 a. m. the horse vomited—without difficulty—a small quantity of fluid which came down the nostrils. At 7.15 he vomited a larger quantity, which as before came down the nostrils, bringing with it, however, a small *Ascaris megalocephala.*

From this time onwards the horse was quiet and apparently out of pain, standing with his head over the half-door of the box, *vomiting frequently;* these acts were accomplished with very little effort,—a slight contraction of the abdominal and cervical muscles accompanied with an elevation of the chin, being all that was noticeable.

A large quantity of fluid was voided in this manner; at times it was passed in a copious gush, at others more gently. The colour was brown, with a gastric odour, more or less masked by the medicines which had been given. Small quantities of chopped hay were co-mingled with the vomit, but the noticeable feature was the presence of ascarides before mentioned; in all twenty-seven were thus ejected, their length varying from 2½ to 12 inches. The horse stood to the last; fell, and died quietly at 4.30 p. m. on December 2nd.

The pulse could not be felt at the jaw after 11 a.m. At 1 p.m. the temperature was 99.8°. The extremities and surface of the body remained cold until death. The conjunctiva was scarlet in colour until a little before death, when it became much less marked.

*Post-mortem* examination, 11 a. m., December 3d. Much gaseous distention of abdomen; a portion of rectum protruding beyond anus; parietal peritoneum red in colour, partly, I think, due to staining, and partly due to inflammatory action. A quantity of sanguineous fluid was in the abdominal cavity. The intestines were of a deep red colour on their outer surface. The stomach was large, red as the intestines on outer portion; contained about a gallon of pultaceous ingesta; the villous portion was congested, but presented no eroded or excoriated patches; there were only a few ascarides in the stomach or intestines.

At a distance of fifteen feet from the pylorus a rent eighteen inches in length was present; the bowel at this part was dark red, almost black, in colour, soft in texture, and much thickened; this condition extended to the cæcum caput coli. The substance of the cæcum was in a similar state to that of the small intestines; its cavity was partially filled with a dark red grumous fluid.

The colon was inflamed and much congested, extravasated blood being mixed with its contents. The results of the inflammatory process were least marked at the commencement of the duodenum. Other organs appeared healthy.

I reserved portions of the affected viscera and their contents, and advised the owner to have them analysed, but this was not done. —*The Veterinarian.*

